# Association between health risks and frailty in relation to the degree of housing damage among elderly survivors of the great East Japan earthquake

**DOI:** 10.1186/s12877-018-0828-x

**Published:** 2018-06-04

**Authors:** M. Tsubota-Utsugi, Y. Yonekura, K. Tanno, M. Nozue, H. Shimoda, N. Nishi, K. Sakata, S. Kobayashi, for the RIAS study

**Affiliations:** 1 Department of Hygiene and Preventive Medicine, Iwate Medical University School of Medicine, 2-1-1 Nishitokuta, Yahaba-cho, Shiwa-gun, Iwate, 028-3694 Japan; 2 Graduate School of Nursing Science, St. Luke’s International University, Tokyo, Japan; 3 Department of Health and Nutritional Sciences, Faculty of Health Promotional Sciences, Tokoha University, Shizuoka, Japan; 4 National Institute of Health and Nutrition, National Institutes of Biomedical Innovation, Health and Nutrition, Tokyo, Japan; 5 Iwate Medical University School of Medicine, Iwate, Japan

## Abstract

**Background:**

Many survivors of the Great East Japan Earthquake that occurred in 2011 were at risk of deteriorating health, especially elderly people living in disaster-stricken areas. The objectives of this prospective study were: a) to clarify the different lifestyle and psychosocial factors associated with frailty by sex among the non-disabled elderly survivors, and b) to describe the differences in characteristics stratified by the degree of disaster-related housing damage.

**Methods:**

We followed 2261 Japanese survivors aged ≥65 years (45.3% male; mean age, 71.7 years) without disability or frailty who completed a self-administered questionnaire at baseline. All participants completed a baseline questionnaire in 2011 and at least one identical follow-up questionnaire between 2012 and 2015 regarding lifestyle (smoking status, alcohol intake, physical activity, sedentary lifestyle, and dietary intake) and psychosocial factors (self-rated health, standard of living, psychological distress, and social networks). Frailty was defined as a score of ≥5 on the Kihon Checklist, which is used by the Japanese government to certify the need for long-term care insurance. Adjusted odds ratios and 95% confidence intervals with frailty as the dichotomous dependent variable and health factors as the independent variables were calculated using a multilevel model for repeated measures by sex, followed by stratification analyses by the degree of housing damage.

**Results:**

Over the 4-year study period, 510 participants (22.6%) developed frailty. In the post-disaster setting, many of the psychosocial factors remained more prevalent 4 years later among survivors with extensive housing damage. The presence of risk factors regarding the development of frailty differed by the degree of housing damage. Among men, psychological distress, in parallel with a poor social network, was related to frailty among only the participants with extensive housing damage and those living in temporary housing, whereas among women, worsening psychological distress was associated only with no damage and no displaced survivors. Among women with extensive damage and displacement, health outcomes such as overweight and diabetes and poor social networks were strongly related to frailty.

**Conclusions:**

Lifestyle and psychosocial factors associated with the risk of frailty differ by sex and the degree of housing damage.

**Electronic supplementary material:**

The online version of this article (10.1186/s12877-018-0828-x) contains supplementary material, which is available to authorized users.

## Background

Frailty is theoretically considered a clinically recognizable state of increased vulnerability resulting from aging [1, 2] and is often synonymous with disability and comorbidity. Frailty is also thought to have an enormous effect on future deterioration in activities of daily living (ADL), disability, hospitalization, and death [3]. To date, a number of lifestyle and psychological factors have been identified as risks of frailty among the community-dwelling elderly [4–9]. In general, older age, low educational level, sedentary lifestyle, obesity, underweight, poor cognitive function, and a history of diseases such as hypertension or depression are associated with the development of frailty; however, definitions of frailty vary widely [4–9].

In March 2011, a massive earthquake and tsunami caused extensive damage in eastern Japan [10]. All survivors were at risk of deteriorating health [11–17], and the elderly were at particularly high risk with regard to disability [12] and cognitive decline [16]. A previous study indicated that the disability prevalence in coastal disaster areas was almost three times higher than that in non-disaster areas [12]. Furthermore, further deterioration of physical and mental functions has been seen among survivors who experienced substantial housing damage owing to the disaster or who were forced to live in places other than their own homes [13, 17]. The degree of housing damage experienced by survivors could be associated with the ongoing effects of the disaster, including displacement. The extant literature examining health effects after a natural disaster have mainly used data from a single time point and focused on survivors living in temporary housing [14, 18, 19]. Moreover, no studies have clarified the differences in lifestyle and psychosocial factors associated with frailty according to the impact of disaster damage. Unlike basic ADL, which consist of self-care tasks such as bathing and dressing, frailty reflects lifestyle factors as well as psychological and social aspects [15, 20]. Identifying specific lifestyle and health conditions associated with frailty in relation to the degree of disaster-related housing damage could be expected to help identify the need for support and encouragement among elderly survivors.

Therefore, the objectives of this prospective study were: a) to clarify the different lifestyle and psychosocial factors associated with frailty by sex among non-disabled elderly survivors of the Great East Japan Earthquake (GEJE), and b) to describe the differences in characteristics stratified by the degree of disaster-related housing damage among survivors of the GEJE in Iwate Prefecture, Japan.

## Methods

### Study design

The present study was part of the Research project for prospective Investigation of health problems Among Survivors of the GEJE (RIAS), a longitudinal observational health study of the devastated area. The details of the RIAS study have been reported previously [13–15]. The survey was conducted as part of general health checkups and employed a common questionnaire inquiring about health conditions and lifestyles. The health checkups were originally part of the national health checkup system and were based on the Regulation Act on Assurance of Medical Care for Elderly People of 1982, which provides six health services, including free general health checkups, to those who reside anywhere in Japan and do not have access to other health examinations such as those provided by their workplace [21]. In the RIAS study, both the eligible and ineligible participants of the original health checkup program were permitted to receive annual health checkups. Data collection was carried out between September 2011 and February 2012 in Yamada town, Otsuchi town, Kamaishi city, and Rikuzentakata city, which were all heavily damaged by the GEJE, in Iwate Prefecture, which is located in the Tohoku area in the northern part of Honshu, Japan’s largest island. We sent out notifications regarding the health survey and questionnaires to all residents aged ≥18 years in Yamada town, Otsuchi town, and Rikuzentakata city; however, in Kamaishi city, we only sent notifications to residents of temporary housing in the Heita area. Using these notifications, we asked residents to complete a questionnaire and return it to their municipal health checkup site. When the residents underwent a health checkup, we explained the study in detail. If the responses in the questionnaires were incomplete, a trained interviewer asked the respondents to answer as completely as possible. Follow-up surveys were repeated annually using similar methods. This study was approved by the Institutional Review Board of Iwate Medical University (approval reference number: H23–69).

### Study population

**Fig. 1:**
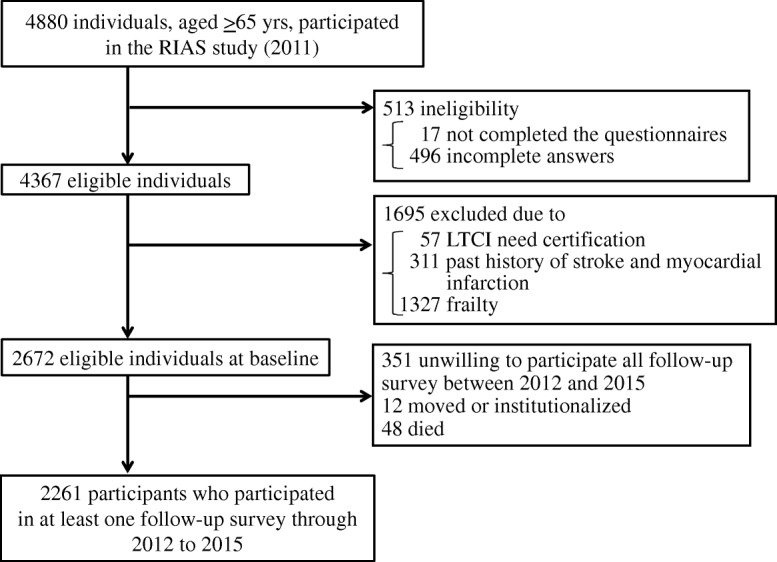
Flow diagram of study participants in the present analysis of the RIAS study, 2011–2015, Japan, LTCI, long-term care insurance; RIAS, Research project for prospective Investigation of health problems Among Survivors of the Great East Japan Earthquake

Figure 1 presents a flow diagram of the study. A total of 11,123 people underwent health checkups, and 10,475 participated in the RIAS study (participation rate, 94.2%). Of the 10,475 participants aged ≥18 years, the final participants were 4880 individuals aged ≥65 years who provided written informed consent for participation and publication in the RIAS study at baseline. The number of participants aged ≥65 years (response rate calculated from the population described in the basic resident register on March 31, 2011 [22]) from each area was 1219 in Yamada town (21.1%), 996 in Otsuchi town (19.7%), 2528 in Rikuzentakata city (31.3%), and 137 in Kamaishi city (we could not calculate the response rate in Kamaishi city because only residents of temporary housing in the Heita area were recruited). Overall, 2208 participants were excluded for the following reasons: incomplete answers on the questionnaire (*n* = 17); missing values in the items assessing the degree of housing damage, frailty, lifestyle, and psychosocial factors (*n* = 496); certification of long-term care insurance (LTCI) needed at baseline (*n* = 57); past history of stroke or myocardial infarction (*n* = 311); and frailty (≥5 points on the Kihon Checklist (KCL), which is used by the Japanese government to certify the need for LTCI (*n* = 1327). Of the remaining 2672 eligible participants at follow-up, we limited the original pool of participants to those who participated in at least one follow-up survey between 2012 and 2015, which resulted in the inclusion of 2261 participants (45.3% male; mean age, 71.7 years). Compared with participants in the follow-up study, the non-participants were more likely to be current smokers (participants vs. non-participants: 9.2% vs. 13.2%) and had a higher prevalence of underweight (1.3% vs. 3.2%), hypertension (59.1% vs. 64.0%), and diabetes mellitus (11.8% vs. 17.7%). However, age and other demographic, lifestyle, and psychosocial factors did not differ significantly between the participants and non-participants (data not tabulated). Among the participants in the baseline RIAS survey, 89.8% of the women, but only 85.8% of the men, participated in the follow-up survey.

### Measures

In the present study, we obtained the variables for sex and the degree of housing damage at one time (baseline), and we used other variables collected during annual checkups, including body mass index (BMI), frailty, and lifestyle and psychosocial factors.

#### Frailty

The risk of frailty was evaluated using the KCL, a multidimensional 20-item index concerning lifestyle, motor abilities, nutrition, oral function, seclusion, and forgetfulness (Additional file 1: Table S1 in the online-only Data Supplement) [23]. The KCL score is useful for predicting the risk of being newly certified as needing LTCI [24, 25]. The response to each item is scored as either 1 (yes) or 0 (no), for a maximum score of 20 points. In the present study, participants whose total KCL score was ≥5 were defined as being frail. KCL scores show good concurrent validity when compared with the Fried frailty criteria [2]. Using a cutoff point between < 5 and ≥ 5, KCL items 0–20 had a sensitivity of 72.2% and a specificity of 80.0% for the Fried frailty criteria [26].

#### Degree of housing damage

Information regarding the degree of housing damage and the accompanying changes in residential status was obtained from the questionnaires. The degree of housing damage was assessed by asking the participants to respond to the question “What was the degree of housing damage due to the disaster?” using one of the following seven options: completely destroyed, large-scale partially destroyed, moderate- to small-scale partially destroyed, partly damaged, not destroyed but flooded, and not destroyed or flooded. On the basis of these answers, the participants were categorized into three groups: extensive (completely and large-scale partially destroyed), partial (moderate- to small-scale partially destroyed to not destroyed but flooded), and no damage (not destroyed or flooded). Since the query on residential status changed according to the stage of the disaster, we revised these into three categories—not displaced, temporary housing, or other residence (family, friend, or relative’s house; newly built house after the disaster; rental apartment; and other)—based on the following two questions: “How many times were you displaced after the disaster? (0, 1, 2, or >2 times)” and “Which residence do you mainly live in now?” (e.g., responses obtained in 2011: own home, home of relative or acquaintance, prefabricated temporary housing, evacuation center, rented accommodation or new home, or other residence; responses obtained in 2015: own home, home of relative or acquaintance, prefabricated temporary housing, private temporary housing, rented accommodation, reconstructed in the same place, reconstructed in another place, or other residence). To avoid misclassifications of the participants’ current situations, we used residential status from an updated version employing data from repeated questionnaires from 2011 to 2015.

#### Lifestyle factors

Data on current smoking and alcohol drinking habits (yes/no) were obtained from annual health surveys using the question “Are you a habitual smoker/drinker?”. Data for other lifestyle factors, such as physical activity, sedentary lifestyle, dietary diversity, and psychological and social factors, were obtained from the self-administered questionnaires. We used the validated Japanese version of the physical activity questionnaires [27]. In brief, the questionnaires asked respondents about (1) the frequency of engaging in physical activity at home and at work, (2) the frequency of leaving their residence, and (3) the length of time spent walking each day. Total scores ranging from 1 to 15 were divided into two sets, < 13.5 and ≥ 13.5, equivalent to < 23 and ≥ 23 metabolic equivalents (METs)·h/week, respectively. Respondents answering the question “How many hours do you normally sit or lie down?” with “≥3 h/day” were classified as having a sedentary lifestyle.

To determine dietary intake, we asked the question, “How many times did you eat each food group per day during the previous several days?” For each of the following, respondents noted whether they ate the given food less than once, once, twice, three times, or four times or more: staple foods (rice, bread, noodles); meat; fish and shellfish; eggs; soybean products; vegetables; fruits; dairy products. Respondents were categorized as having good dietary diversity if their responses matched all of the following criteria: (1) staple foods ≥3 times/day; (2) the same or a combination of protein-related food groups (meat, fish and shellfish, eggs, and soybean products) ≥2 times/day; (3) vegetables ≥2 times/day; (4) fruits ≥1 time/day; and (5) dairy products ≥1 time/day [28].

#### Psychosocial factors

Self-rated health (SRH) was assessed by asking, “How do you feel about your health condition?” with the following four options: very good, good, not very good, and not good. On the basis of the respondents’ answers, four options were categorized into two groups: good (very good and good) and not good (not very good and not good). To assess standard of living, we asked the participants, “How do you feel about your current economic situation?”. We divided the four response options into two groups: difficult (severely difficult, difficult, and slightly difficult) and acceptable. Kessler’s 6-item psychological distress scale (K6) was used to assess psychological distress [29]. The K6 is composed of six questions regarding how often an individual has felt the following in the previous month: nervous, hopeless, restless or fidgety, so sad that nothing could cheer them up, everything requires effort, and worthless. The responses ranged from never (0 points) to all of the time (4 points). Scores of > 10/24 points were considered to indicate the presence of psychological distress [30, 31].

Social networks were assessed using the 6-item Lubben Social Network Scale [32, 33]. The reproducibility and validity of the Japanese version have been reported in detail [32]. The questionnaire asked about the numbers of relatives or friends in response to the following questions: “How many relatives/friends do you see or hear from at least once a month?”, “How many relatives/friends do you feel close to such that you could call on them for help?”, and “How many relatives/friends do you feel at ease with such that you could talk with them about private matters?”. The responses consisted of five frequency categories ranging from none (0 points) to nine or more (5 points), and the total scores ranged from 0 to 30. We classified participants with scores of < 12/30 points as having a poor social network.

#### Other factors

Other factors, including present job (none or unemployed, yes without any changes, and yes with changes owing to the disaster), BMI (weight kg/m^2^), hypertension, diabetes mellitus, and hypercholesterolemia, were also taken into consideration. BMI was calculated, and each participant was classified as underweight (< 18.5 kg/m^2^), normal weight (18.5–24.9 kg/m^2^), or overweight (≥25.0 kg/m^2^). Blood pressure was measured twice consecutively in a sitting position using an automatic device after urination and a 5-min rest period. Hypertension was defined as the use of antihypertensive medication and/or blood pressure ≥ 140/90 mmHg. We examined the results of blood tests to determine the total cholesterol (TC) level (mg/dL) using an automated analyzer. Glycosylated hemoglobin (HbA1c) (Japan Diabetes Society [JDS] in 2011 and 2012 and the National Glycohemoglobin Standardization Program [NGSP] from 2012, %) was determined using an automated analyzer. The HbA1c value (%) was estimated as the NGSP equivalent value (%), which was calculated using the following formula: HbA1c (NGSP)(%) = HbA1c (JDS) (%) + 0.4 (%) [34]. Diabetes mellitus was defined as a random blood glucose level ≥ 11.11 mmol/L (≥200 mg/dL), HbA1c ≥6.5% (NGSP), and/or the use of medications for diabetes. Hypercholesterolemia was defined as TC ≥5.68 mmol/L (≥220 mg/dL) and/or the use of medication for hypercholesterolemia.

### Statistical analyses

All analyses were performed separately for men and women because the distribution of lifestyle and psychological characteristics differed by sex (Table 1). The Student t-test for continuous variables and the chi-square test for categorical variables were used to evaluate the baseline characteristics of participants by sex who experienced frailty during follow-up. Using repeated data on lifestyle and psychosocial factors from each follow-up survey, Cochran–Mantel–Haenszel tests for trends were used to assess differences in characteristics across the degree of housing damage and adjusted for age among the participants for all 5 years (*n* = 1529).

**Table 1:** Baseline characteristics of the participants by sex, RIAS study, 2011

	Male (*n* = 1025)			Female (*n* = 1236)			*P*-value^b^
	Non-frailty	Frailty	*P*-value^a^	Non-frailty	Frailty	*P*-value^a^	
Number of participants	813	212		938	298		
Age, mean ± SD	71.9 ± 4.7	73.7 ± 5.0	*<.001*	70.9 ± 4.4	72.7 ± 5.1	*<.001*	*<.001*
Age, ≥75 years %	27.3	40.1	*<.001*	19.7	32.6	*<.001*	*<.001*
The degree of housing damage, extensive %	38.9	42.0	*0.492*	40.8	41.3	*0.367*	*0.226*
Job without change due to the disaster, %	21.0	22.0	*0.166*	20.4	19.1	*0.827*	*<.001*
Job with change due to the disaster, %	24.9	30.9		19.8	19.1		
Residential status, temporary %	20.4	21.7	*0.109*	20.8	22.3	*0.842*	*0.092*
Residential status, other residence %	22.2	28.3		27.3	27.4		
BMI, mean ± SD	24.1 ± 2.7	24.2 ± 2.9	*0.406*	23.5 ± 3.2	24.0 ± 3.5	*0.014*	*<.001*
BMI, normal %	64.4	62.8	*0.329*	70.0	58.7	*0.001*	*<.001*
BMI, underweight %	0.3	0.9		2.1	1.7		
BMI, overweight %	35.3	36.3		27.9	39.6		
Hypertension, %	62.2	60.4	*0.619*	55.8	60.4	*0.158*	*0.017*
Diabetes mellitus, %	14.5	18.4	*0.193*	7.8	11.4	*0.052*	*<.001*
Hypercholesterolemia, %	25.7	26.4	*0.834*	36.3	32.6	*0.245*	*<.001*
Current smokers, %	19.2	18.9	*0.963*	1.1	1.0	*0.075*	*<.001*
Current drinkers, %	40.7	39.6	*0.932*	1.5	1.3	*0.026*	*<.001*
Physical activity, <23METs·hour/week %	55.8	58.7	*0.451*	62.7	69.7	*0.028*	*<.001*
Sedentary lifestyle, %	27.6	31.6	*0.259*	26.4	31.3	*0.097*	*0.664*
Poor dietary diversity, %	43.5	50.9	*0.052*	29.6	42.3	*<.001*	*<.001*
Self-rated health, poor %	7.0	14.6	*0.001*	7.8	17.5	*<.001*	*0.208*
Standard of living, difficult %	37.5	42.1	*0.221*	36.3	41.9	*0.083*	*0.701*
Psychological distress, %	4.5	10.1	*0.002*	8.9	14.3	*0.008*	*<.001*
Poor social networks, %	28.8	47.3	*<.001*	26.0	35.3	*0.002*	*0.027*

^a^Obtained using the t-test for continuous variables and the chi-square test for categorical variables, comparing non-frailty with frailty by sex

^b^Obtained using the t-test for continuous variables and the chi-square test for categorical variables, comparing male participants (*n* = 1025) with female participants (*n* = 1236)

To clarify the factors associated with frailty by sex, multilevel regression models with occasions of measurement nested within individuals were employed to model individual-level changes in frailty over time, measured as years since baseline [35, 36]. BMI and lifestyle and psychosocial factors were used as time-varying covariates over time [Level 1], while baseline age and sex were entered as time-fixed covariates of inter-individual variations in frailty [Level 2]. A two-step process was used to determine the best model for the study. First, we selected variables based on plausibility and the literature. Second, to examine how each variable independently affected the odds ratios, an age-adjusted analysis was conducted to determine the associations of all separately added variables with frailty in the present study. Finally, all variables showing *P* ≤ .10 in an age-adjusted analysis were considered for inclusion in the final models: present job, BMI, diabetes mellitus, current drinking habits, physical activity, sedentary lifestyle, dietary diversity, SRH, standard of living, psychological distress, and social network for male participants; and present job, BMI, diabetes mellitus, sedentary lifestyle, dietary diversity, SRH, standard of living, psychological distress, and social network for female participants. Next, to assess the difference of the effect according to the degree of housing damage, we stratified the participants by the degree of housing damage and current residential status and tested for interactions between the degree of housing damage and residential status and lifestyle and psychosocial factors to determine the associations with frailty.

In addition, we performed a sensitivity analysis to examine the influence of preclinical cases of disability during follow-up; participants who were certified as needing LTCI during follow-up were excluded [37, 38]. To account for the fact that the follow-up time would be different for participants with no data in the later waves, an additional analysis with only participants who participated in all health checkups from 2011 through 2015 was conducted. Possible interactions were tested by introducing a multiplicative term into the main effect models.

For all analyses, statistical significance was defined as an α level of <.05 for two-sided tests. All statistical analyses were performed using SAS software (version 9.4; SAS Institute, Cary, NC, USA).

## Results

### Participants’ characteristics

Over the 4 years after the disaster from 2011 to 2015, 510 participants (22.6%) developed frailty, and 71 (3.1%) were certificated as needing LTCI for the first time. The proportions of the development of frailty by 2015 in relation to the degree of housing damage were as follows: male 19.0%/female 25.2% for no damage, male 22.1%/female 20.0% for partial damage, and male 22.0%/female 24.3% for extensive damage. Compared with those who had experienced no or partial damage, the vast majority of those who had experienced extensive housing damage were still displaced from their own homes: 94.5% of the males and 96.2% of the females who had experienced extensive housing damage were displaced because of the GEJE and subsequent tsunami, and 52.0% of the males and 49.8% of the females were living in temporary housing. Only women living in temporary housing were older than those with “other” residential status (71.7 vs. 70.7 years old, respectively, *P* = 0.030). Table 1 shows the baseline characteristics of the participants according to frailty by sex. Compared with the male participants, females tended to be younger, to have a lower BMI, to less frequently be current smokers or drinkers, and to have a lower prevalence of hypertension and diabetes; however, they also showed a higher prevalence of hypercholesterolemia and psychological distress. Both men and women who developed frailty were older and had a higher prevalence of poor SRH, psychological distress, and poor social networks than those who did not. Compared with those who did not, females who developed frailty had a higher prevalence of overweight, physical inactivity, and poor dietary diversity, and a lower prevalence of underweight.

### Changes in lifestyle and psychosocial factors from 2011 to 2015

**Fig. 2:**
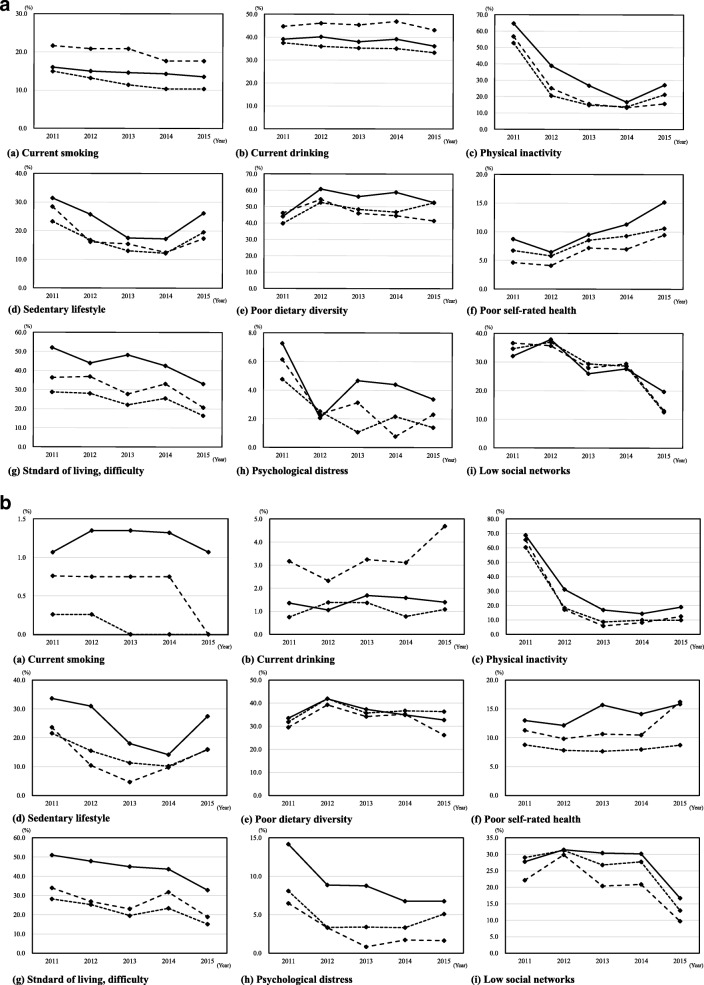
**a** Age-adjusted changes in lifestyle and psychosocial factors by degree of housing damage^†^ in men (*n* = 679). ^†^Degree of housing damage: extensive damage (solid line); partial damage (dashed line); and no damage (dotted line). All factors a) through i), *p* < .001 by Cochran–Mantel–Haenszel tests for trends adjusted for age. A significant result on this test means that after controlling for age and time of year, the proportion of people who reported a certain factor was different depending on the degree of housing damage. **b** Age-adjusted changes in lifestyle and psychosocial factors by degree of housing damage^†^ in women (*n* = 850). ^†^Degree of housing damage: extensive damage (solid line); partial damage (dashed line); and no damage (dotted line). Factors c) through i), *p* < .001 by Cochran–Mantel–Haenszel tests for trends adjusted for age. A significant result on this test means that after controlling for age and time of year, the proportion of people who reported a certain factor was different depending on the degree of housing damage

Age-adjusted changes in lifestyle and psychosocial factors from 2011 to 2015 stratified by sex and degree of housing damage are shown in Fig. 2a for men and b for women. As expected, survivors who had experienced extensive damage showed a significantly higher proportion of health deteriorations within the whole period compared with those who had experienced no damage (*P* < .001 by Cochran–Mantel–Haenszel tests for trends). Four years after the disaster, physical inactivity, sedentary lifestyle, a difficult standard of living, poor SRH, psychological distress, and a poor social network in both sexes, and poor dietary diversity in men, were still more frequent among survivors who had experienced extensive damage compared with those who had experienced partial or no damage. On the contrary, physical inactivity, a difficult standard of living, psychological distress, and a poor social network gradually improved after the disaster for all degrees of housing damage. In particular, those who initiated physical inactivity markedly decreased from 2011 to 2012 for all degrees of housing damage in both sexes (change in physical inactivity from 2011 to 2012; Fig. 2a-(c) for men; no damage, 52.8 to 20.5%: partial damage, 56.7 to 25.1%: and extensive damage, 64.7 to 38.8%; Fig. 2b-(c) for women; no damage, 60.3 to 18.2%: partial damage, 65.6 to 17.2%: and extensive damage, 68.7 to 31.2%). Similar declines for lifestyle and psychosocial risks during the first year were seen in sedentary lifestyle and psychological distress, whereas poor dietary diversity in men and poor SRH increased in the follow-up period (2011–2015).

### Associations between lifestyle and psychosocial factors and the development of frailty

Table 2 shows the association between lifestyle and psychosocial factors and the development of frailty. Overall, males with poor dietary diversity (odds ratio [95% confidence interval], 1.61 [1.23–2.09]), poor SRH (2.94 [2.17–3.98]), psychological distress (2.15 [1.15–4.02]), and a poor social network (1.82 [1.38–2.41]), and females classified as overweight (1.42 [1.10–1.82]), having diabetes mellitus (1.46 [1.04–2.03]), a sedentary lifestyle (1.54 [1.20–1.98]), poor dietary diversity (1.49 [1.19–1.87]), poor SRH (3.03 [2.24–4.09]), a difficult standard of living (1.33 [1.04–1.71]), and a poor social network (1.33 [1.06–1.67]) were associated with an increased risk for the development of frailty. These significant associations were also observed in the linear regression models, except for diabetes mellitus in women (Additional file 2**:** Table S2–1 for men and Additional file 3**:** Table S2–2 for women in the online-only Data Supplement). In the present study, the results from an additional analysis conducted with only participants who participated in all health checkups from 2011 through 2015 did not alter these associations, except for diabetes mellitus in women.

**Table 2:** Factors associated with the onset of frailty^a^ among elderly male and female survivors in the RIAS study, 2011–2015

Variable	Male				Female			
	All participants in the present study.		Only those who participated in all from 2011 to 2015.		All participants in the present study.	Only those who participated in all from 2011 to 2015.		
Number	1025		679		1236	850		
*Adjusted OR (95% CI) P-value*								
BMI, underweight	2.66 (0.84–8.45)	*0.098*	2.22 (0.47–10.57)	*0.317*	1.17 (0.54–2.52)	*0.692*	1.02 (0.45–2.33)	*0.959*
BMI, overweight	1.08 (0.81–1.44)	*0.612*	1.35 (0.94–1.96)	*0.108*	1.42 (1.10–1.82)	*0.006*	1.37 (1.02–1.83)	*0.038*
Diabetes mellitus	1.16 (0.83–1.62)	*0.388*	1.23 (0.80–1.90)	*0.338*	1.46 (1.04–2.03)	*0.027*	1.25 (0.82–1.90)	*0.298*
Sedentary lifestyle	1.12 (0.84–1.50)	*0.447*	1.08 (0.74–1.59)	*0.680*	1.54 (1.20–1.98)	*0.001*	1.57 (1.17–2.12)	*0.003*
Poor dietary diversity	1.61 (1.23–2.09)	*<.001*	1.70 (1.22–2.36)	*0.002*	1.49 (1.19–1.87)	*0.001*	1.45 (1.12–1.87)	*0.005*
Poor self-rated health	2.94 (2.17–3.98)	*<.001*	2.83 (1.91–4.21)	*<.001*	3.03 (2.24–4.09)	*<.001*	3.14 (2.20–4.50)	*<.001*
Standard of living, difficult	1.28 (0.97–1.70)	*0.082*	1.54 (1.07–2.20)	*0.019*	1.33 (1.04–1.71)	*0.022*	1.35 (1.01–1.79)	*0.040*
Psychological distress	2.15 (1.15–4.02)	*0.017*	3.10 (1.53–6.26)	*0.002*	1.43 (0.98–2.08)	*0.065*	1.35 (0.85–2.13)	*0.204*
Poor social networks	1.82 (1.38–2.41)	*<.001*	1.67 (1.15–2.42)	*0.007*	1.33 (1.06–1.67)	*0.015*	1.36 (1.04–1.77)	*0.024*

*BMI* body mass index, *OR* odds ratio, *95% CI* 95% confidence interval

Non-significant variables are not displayed in table

Adjusted for males: age, BMI (underweight: < 18, overweight: ≥25 vs. normal: 18–25 kg/m^2^), diabetes mellitus (yes vs. no), current drinker (yes vs. no), physical activity (inactivity: < 23 METs·h/week vs. ≥23 METs·h/week), sedentary lifestyle (yes vs. no), poor dietary diversity (yes vs. no), poor self-rated health (yes vs. no), standard of living (difficult vs. acceptable), psychological distress (yes vs. no), and poor social network (yes vs. no); females: age, BMI (underweight: < 18, overweight: ≥25 vs. normal: 18–25 kg/m^2^), diabetes mellitus (yes vs. no), sedentary lifestyle (yes vs. no), poor dietary diversity (yes vs. no), poor self-rated health (yes vs. no), standard of living (difficult vs. acceptable), psychological distress (yes vs. no), and poor social network (yes vs. no)

^a^Survivors were classified into two groups: frailty (≥5 points on the Kihon Checklist, which is used by the Japanese government to certify the need for long-term care insurance) and non-frailty (< 5 points on the Kihon Checklist)

### Characteristic differences related to the development of frailty by degree of housing damage

Next, we conducted stratification analyses according to the degree of housing damage and residential status (Table 3 for men and Table 4 for women). Although no significant interactions were found between the degree of damage and any of the other variables in regard to associations with the development of frailty in both sexes (P for interaction ≥.2), noteworthy differences in characteristics were seen in relation to the development of frailty by the degree of housing damage.

**Table 3:** Factors associated with the onset of frailty^a^ stratified by the degree of housing damage among elderly male survivors in the RIAS study, 2011–2015

Variable	*By the degree of housing damage*						*By residential status* ^*b*^					
	No		Partial		Extensive		No displaced		Temporary		Other residence	
Number	448		172		405		603		176		246	
*Adjusted OR (95% CI) P-value*												
BMI, underweight	0.90 (0.09–8.74)	*0.931*	15.80 (0.74–337.16)	*0.077*	3.53 (0.74–16.92)	*0.114*	1.88 (0.34–10.42)	*0.468*	11.85 (5.35–26.23)	*<.001*	3.78 (0.34–41.96)	*0.279*
Current drinkers	0.83 (0.51–1.38)	*0.475*	0.73 (0.38–1.43)	*0.364*	0.77 (0.47–1.27)	*0.309*	0.82 (0.53–1.26)	*0.361*	0.76 (0.34–1.67)	*0.486*	0.79 (0.46–1.36)	*0.388*
Physical inactivity	0.94 (0.63–1.42)	*0.771*	1.03 (0.54–1.96)	*0.922*	1.43 (0.96–2.12)	*0.077*	0.99 (0.69–1.41)	*0.939*	0.89 (0.47–1.66)	*0.706*	1.64 (1.03–2.62)	*0.039*
Sedentary lifestyle	1.26 (0.79–2.01)	*0.325*	1.06 (0.55–2.02)	*0.864*	1.02 (0.64–1.61)	*0.943*	1.14 (0.76–1.72)	*0.522*	0.89 (0.42–1.87)	*0.749*	1.08 (0.64–1.82)	*0.770*
Poor dietary diversity	1.43 (0.94–2.18)	*0.092*	2.43 (1.26–4.69)	*0.008*	1.54 (1.02–2.32)	*0.042*	1.66 (1.15–2.39)	*0.006*	0.75 (0.42–1.36)	*0.348*	2.15 (1.27–3.62)	*0.004*
Poor self-rated health	3.62 (2.25–5.83)	*<.001*	2.60 (1.09–6.16)	*0.031*	2.42 (1.54–3.81)	*<.001*	3.39 (2.20–5.21)	*<.001*	4.68 (2.49–8.80)	*<.001*	1.74 (0.95–3.19)	*0.075*
Standard of living, difficult	1.69 (1.11–2.59)	*0.015*	1.22 (0.64–2.33)	*0.546*	1.15 (0.74–1.78)	*0.525*	1.51 (1.04–2.19)	*0.029*	0.88 (0.43–1.80)	*0.729*	1.32 (0.80–2.20)	*0.276*
Psychological distress	1.10 (0.32–3.80)	*0.882*	1.58 (0.32–7.72)	*0.571*	3.23 (1.42–7.37)	*0.005*	1.22 (0.45–3.34)	*0.698*	7.98 (3.63–17.54)	*<.001*	1.61 (0.38–6.86)	*0.517*
Poor social networks	1.64 (1.01–2.65)	*0.045*	1.45 (0.74–2.86)	*0.279*	2.21 (1.46–3.37)	*<.001*	1.68 (1.13–2.49)	*0.011*	2.42 (1.39–4.20)	*0.002*	2.17 (1.29–3.65)	*0.004*

*BMI* body mass index, *OR* odds ratio, *95% CI* 95% confidence interval

Non-significant variables are not displayed

Adjusted for age, BMI (underweight: < 18, overweight: ≥25 vs. normal: 18–25 kg/m^2^), diabetes mellitus (yes vs. no), current drinker (yes vs. no), physical activity (inactivity: < 23 METs·h/week vs. ≥23 METs·h/week), sedentary lifestyle (yes vs. no), poor dietary diversity (yes vs. no), poor self-rated health (yes vs. no), standard of living (difficult vs. acceptable), psychological distress (yes vs. no), and poor social network (yes vs. no)

^a^Survivors were classified into two groups: frailty (≥5 points on the Kihon Checklist, which is used by the Japanese government to certify the need for long-term care insurance) and non-frailty (< 5 points on the Kihon Checklist)

^b^To avoid misclassifying participants’ current situations, we continued updating each person’s residential status throughout the follow-up period using data from repeated questionnaires

**Table 4:** Factors associated with the onset of frailty^a^ stratified by the degree of housing damage among elderly female survivors in the RIAS study, 2011–2015

Variable	*By the degree of housing damage*						*By residential status* ^*b*^					
	No		Partial		Extensive		No displaced		Temporary		Other residence	
Number	555		175		506		694		262		280	
*Adjusted OR (95% CI) P-value*												
BMI, underweight	2.30 (1.03–5.14)	*0.043*	1.42 (0.21–9.40)	*0.719*	0.42 (0.08–2.34)	*0.323*	2.29 (1.08–4.86)	*0.031*	0.65 (0.10–4.37)	*0.654*	0.36 (0.03–3.97)	*0.403*
BMI, overweight	1.16 (0.80–1.68)	*0.435*	1.68 (0.80–3.56)	*0.173*	1.70 (1.15–2.51)	*0.008*	1.33 (0.96–1.86)	*0.091*	1.64 (0.96–2.81)	*0.073*	1.50 (0.87–2.60)	*0.147*
Diabetes mellitus	1.53 (0.94–2.49)	*0.091*	0.47 (0.17–1.26)	*0.134*	1.82 (1.09–3.04)	*0.023*	1.13 (0.73–1.77)	*0.583*	1.77 (0.91–3.44)	*0.094*	2.40 (1.07–5.38)	*0.034*
Sedentary lifestyle	1.64 (1.10–2.44)	*0.016*	1.61 (0.68–3.81)	*0.276*	1.49 (1.04–2.13)	*0.029*	1.49 (1.05–2.11)	*0.026*	2.08 (1.32–3.29)	*0.002*	1.45 (0.82–2.57)	*0.199*
Poor dietary diversity	1.52 (1.10–2.08)	*0.010*	1.30 (0.62–2.72)	*0.479*	1.56 (1.09–2.25)	*0.016*	1.47 (1.09–1.98)	*0.011*	1.42 (0.90–2.23)	*0.131*	1.55 (0.93–2.60)	*0.094*
Poor self-rated health	3.20 (1.97–5.19)	*<.001*	2.29 (1.01–5.18)	*0.046*	3.57 (2.31–5.53)	*<.001*	2.70 (1.81–4.05)	*<.001*	3.12 (1.54–6.32)	*0.002*	4.29 (2.37–7.77)	*<.001*
Standard of living, difficult	1.55 (1.08–2.24)	*0.019*	1.97 (0.91–4.24)	*0.084*	1.17 (0.80–1.71)	*0.412*	1.42 (1.00–2.01)	*0.052*	1.76 (1.09–2.85)	*0.021*	0.99 (0.56–1.75)	*0.968*
Psychological distress	1.95 (1.13–3.38)	*0.017*	0.86 (0.19–3.88)	*0.840*	1.35 (0.79–2.28)	*0.270*	1.89 (1.09–3.28)	*0.024*	0.90 (0.48–1.70)	*0.750*	1.89 (0.79–4.52)	*0.155*
Poor social networks	1.24 (0.91–1.71)	*0.176*	1.45 (0.68–3.09)	*0.330*	1.44 (0.99–2.08)	*0.054*	1.34 (1.00–1.79)	*0.053*	1.06 (0.66–1.72)	*0.809*	1.85 (1.07–3.20)	*0.027*

*BMI* body mass index, *OR* odds ratio, *95% CI* 95% confidence interval

Non-significant variables are not displayed

Adjusted for age, BMI (underweight: < 18, overweight: ≥25 vs. normal: 18–25 kg/m^2^), diabetes mellitus (yes vs. no), sedentary lifestyle (yes vs. no), poor dietary diversity (yes vs. no), poor self-rated health (yes vs. no), standard of living (difficult vs. acceptable), psychological distress (yes vs. no), and poor social network (yes vs. no)

^a^Survivors were classified into two groups: frailty (≥5 points on the Kihon Checklist, which is used by the Japanese government to certify the need for long-term care insurance) and non-frailty (< 5 points on the Kihon Checklist)

^b^To avoid misclassifying participants’ current situation, we continued updating each person’s residential status throughout the follow-up period using data from repeated questionnaires

### Associated factors for the development of frailty among residents with extensive housing damage

Among residents with extensive housing damage, poor dietary diversity (1.54 [1.02–2.32]), poor SRH (2.42 [1.54–3.81]), psychological distress (3.23 [1.42–7.37]), and a poor social network (2.21 [1.46–3.37]) among men, and overweight (1.70 [1.15–2.51]), diabetes mellitus (1.82 [1.09–3.04]), sedentary lifestyle (1.49 [1.04–2.13]), poor dietary diversity (1.56 [1.09–2.25]), and poor SRH (3.57 [2.31–5.53]) among women were independently associated with the development of frailty.

These risk factors were similar among those that had been displaced, but several differences in factors were found. Poor SRH (4.68 [2.49–8.80]), psychological distress (7.98 [3.63–17.54]), and a poor social network (2.42 [1.39–4.20]) were strongly related to frailty only among men living in temporary housing, and poor dietary diversity (2.15 [1.27–3.62]) and a poor social network (2.17 [1.29–3.65]) were independently associated with the risk of frailty among men living in other residences. In addition, an association with underweight was seen among men living in temporary housing (11.85 [5.35–26.23]) with a large interval, as was an association with physical inactivity among men living in other residences (1.64 [1.03–2.62]). On the other hand, the associations with overweight and poor dietary diversity disappeared among women who had been displaced. An association between sedentary lifestyle (2.08 [1.32–3.29]) and the risk of frailty was seen among women living in temporary housing, and having diabetes mellitus (2.40 [1.07–5.38]) was observed only among women living in other residences. In addition, an association with a difficult standard of living was seen among women living in temporary housing (1.76 [1.09–2.85]), as was an association with poor social networks among women living in other residences (1.85 [1.07–3.20]).

### Associated factors for the development of frailty among residents with no or partial housing damage

Among residents with no or partial housing damage, the association of poor SRH among men with no or partial housing damage (3.62 [2.25–5.83] for men with no housing damage, 2.60 [1.09–6.16] for men with partial damage), the association of a difficult standard of living (1.69 [1.11–2.59]) and a poor social network (1.64 [1.01–2.65]) among men with no damage, and the association of poor dietary diversity among men with partial housing damage (2.43 [1.26–4.69]), were independently associated with the development of frailty among men. By contrast, in women, an association with poor SRH and the risk of frailty was seen only among those with partial housing damage (2.29 [1.01–5.18]), and many lifestyle and psychosocial factors were associated with the development of frailty among women with no housing damage (underweight: 2.30 [1.03–5.14]; sedentary lifestyle: 1.64 [1.10–2.44]; poor dietary diversity: 1.52 [1.10–2.08]; poor SRH: 3.20 [1.97–5.19]; a difficult standard of living: 1.55 [1.08–2.24]; and psychological distress: 1.95 [1.13–3.38]). These risk factors were analogous to the results among those who had not been displaced among both sexes.

Similar associations were seen in the linear model for men, whereas the specific associations observed only in women who experienced housing damage or been displaced in the logistic regression, i.e., overweight and diabetes, disappeared in the linear model (Additional file 2**:** Table S2–1 for men and Additional file 3**:** Table S2–2 for women in the online-only Data Supplement). An additional sensitivity analysis excluding participants certified as needing LTCI during the follow-up period did not alter the results (data not shown).

## Discussion

In the post-disaster setting, many of the psychosocial factors remained more prevalent 4 years later among survivors who had experienced extensive damage. Among men who had experienced extensive housing damage, poor dietary diversity, poor SRH, psychological distress, and a poor social network were independently associated with the development of frailty, whereas among women who had experienced extensive housing damage, overweight, diabetes mellitus, sedentary lifestyle, poor dietary diversity, and poor SRH were significantly associated with the risk of frailty. These risk factors were also similar to the results regarding the accompanying changes in residential status, but several differences in factors were found between temporary housing and other residences. To the best of our knowledge, this is the first study to examine changes in lifestyle and psychosocial characteristics from 6 months after the GEJE to the present.

### Changes in lifestyle and psychosocial factors from 2011 to 2015

Our findings regarding changes in lifestyle and psychosocial factors from 2011 to 2015 revealed that the number of survivors with physical inactivity and an increased risk of psychosocial factors markedly decreased from 2011 to 2012; however, men with poor dietary diversity and both men and women with poor SRH increased or did not change during the follow-up period. An explanation for this finding is that many survivors recovered from their disaster-induced temporary lifestyle and psychosocial deterioration within 1 or 2 years. On the other hand, even 4 years after the disaster, physical inactivity, sedentary lifestyle, a difficult standard of living, poor SRH, psychological distress, and a poor social network in both sexes, as well as poor dietary diversity in males, remained more prevalent among participants who had experienced extensive housing damage.

### Associations between lifestyle and psychosocial factors and the development of frailty

Overall, in the present study, poor dietary diversity, poor SRH, psychological distress, and a poor social network in males, and overweight, diabetes mellitus, sedentary lifestyle, poor dietary diversity, poor SRH, a difficult standard of living, and a poor social network in females, were associated with the development of frailty in non-disabled older Japanese disaster survivors. These risk factors were also confirmed in the linear model using 1-point increments on the KCL. These results are consistent with findings from previous studies involving community residents [4–9].

### Characteristic differences related to the development of frailty by degree of housing damage

The present study revealed that the presence of risk factors regarding the development of frailty differed by the degree of housing damage, although no significant interactions were observed. Compared with survivors who had experienced no or partial damage, the majority of those who had experienced extensive housing damage were still forced to relocate from their own homes and change their residences.

### Associated factors for the development of frailty among residents with extensive housing damage

In the present study, poor dietary diversity, poor SRH, psychological distress, and a poor social network were associated with a higher risk of the development of frailty among men who had experienced extensive housing damage. These associations with frailty among men who had experienced extensive housing damage were similar to current residential status with displacement, but slightly different between temporary housing and other residences. The factors associated with frailty among men living in temporary housing were underweight, poor SRH, psychological distress, and a poor social network, whereas the risk factors among men living in other residences were physical inactivity, poor dietary diversity, and a poor social network.

On the other hand, among women who had experienced extensive housing damage, overweight, diabetes mellitus, sedentary lifestyle, poor dietary diversity, and poor SRH were associated with the development of frailty; however, overweight and poor dietary diversity with the risk of frailly disappeared among women who had been displaced. Instead, a difficult standard of living among women living in temporary housing was associated with the development of frailty. Many elderly people live on pension and depend on a limited income. Since those living in temporary housing do not have to pay rent or related utility expenses, it was thought that women would still be forced to live in temporary housing. A difficult standard of living is considered the reason that the development of frailty and disability may be accelerated among survivors.

In the present study, a sedentary lifestyle was associated with the risk of frailty among women who had experienced extensive housing damage and those living in temporary housing. Sedentary risk in temporary housing is consistent with findings from a previous study [15]. Compared with other types of residences, temporary housing is constructed on unused or undeveloped land, so no facilities or public transport services are available in the surrounding areas. Therefore, those who were living in temporary housing faced more difficulties in terms of accessing other facilities and transport. Indeed, a previous study reported that the longer the distance to retail stores, the higher the risk of not going out [39]. In addition, people living in temporary housing had only a limited and narrow living space, and thus did not need to move so much to perform daily chores. In the present study, women living in temporary housing were older than those living in other residences. It is possible that the women living in temporary housing stopped going out as a result of being restricted to the surrounding areas. Enabling residents living in temporary housing to access facilities and retail stores might therefore be effective for preventing the sedentary lifestyles related to frailty.

Alternately, among women, an association between frailty and a poor social network was recognized only among those living in other residences. After the GEJE, in anticipation of a delay in construction owing to a lack of land and materials, the Japanese government actively utilized privately rented temporary housing in which municipalities rented out private homes in parallel with the construction of temporary housing [40]. Therefore, among women forced to move a long distance from the disaster area, the difficulty of accessing existing communities and the inability to share their experience of the disaster or damage might have increased the risk of frailty. By contrast, the reason that a risk of frailty was not associated with psychological distress or a poor social network among women living in temporary housing may be explained by the fact that the residents living in temporary housing had experienced the same degree of housing damage. Temporary housing is associated with an increased risk of social isolation [13, 15], but it might also play a significant role in enhancing social networks by moving the same community residents to temporary housing in the same district or by constructing new communities [41]. Previous studies have implied that the presence of social connections in temporary housing communities, e.g., facilitating social networks and building social capital, is associated with increased physical activity such as walking duration and time outside of the home, as well as the prevention of psychological distress and cognitive impairment [17, 19, 42]. Considering the different social roles between sexes, the preventive effect of a good social network on frailty in disaster-stricken areas can be expected in women more than in men. Combined with the effect of a poor social network on the risk of frailty among men with any residential status, the effect of disaster-induced psychological distress on male participants who had experienced extensive housing damage or relocated might be more severe compared with that on women. At present, the construction of public housing for those affected by disasters is proceeding. Future research is expected to elucidate the risks associated with lifestyle and psychosocial factors in accordance with differences in more detailed residential environments.

### Associated factors for the development of frailty among residents with no or partial housing damage

Since the proportion of residents who had experienced partial housing damage in the present study was low, the obtained results are not definitively conclusive. However, the present study did reveal that the risk factors in survivors who had experienced no housing damage or had been displaced were similar to findings from previous studies involving community residents [4–9], although the participants in the present study were restricted to being survivors of the GEJE only. Although the reasons for these results remain somewhat unclear, several possible explanations regarding differences in the selection of participants by degree of damage were considered.

As described in the Methods, health checkups were originally part of the national health checkup system based on the Regulation Act on Assurance of Medical Care for Elderly People of 1982, and participants were restricted to those who resided anywhere in Japan and did not have access to other health examinations. However, since the disaster, the RIAS study has been providing free annual health checkups to all survivors who wish to have one. In general, participants who undergo annual health checkups tend to be more conscious about their health [43, 44]. Medical examinations in the workplace are mandatory for employers to provide and for workers to undergo, whereas health checkups are largely the intention of residents themselves. In other words, continuous participants in the RIAS study were more likely to be sufficiently healthy to participate voluntarily, and survivors with serious psychological and/or physical health problems who were living in residences with extensive housing damage could not have participated in health checkups in the first place. In addition, particularly in Japan, where the employment rate among women is relatively low [45], many elderly women were considered to be eligible to participate in the original health checkup program. This is inferred from the fact that many women remain participants in the follow-up survey, in contrast to many men, who only participated for the first year based on a comparison of participants and non-participants. Lastly, most of the factors in this research were self-reported, except for the anthropometric and biochemical examinations. A previous study implied that poor functional status in physical, mental, and social factors, which were mostly self-reported, is associated with the risk of frailty, whereas routine clinical tests such as blood pressure are seldom associated with the development of frailty [5, 22]. Women are more likely to experience physical and social discomfort than men [46, 47]. In the present study, women who had experienced no housing damage may have had more original risk factors than those who had experienced extensive housing damage. Given the above reasons and the present results, it is possible that women eligible for the original health checkup program who had not experienced any housing damage were more likely to participate in the health checkups, which could create a vicious circle leading to the development of further risk factors in the future.

Several caveats must be considered when interpreting our findings. First, because we collected data at health checkups, the participants may not have accurately represented the community in the GEJE area. Since our study population experienced a disaster directly and lived in a disaster-affected area, survivors with serious psychological and/or physical health problems may not have undergone health checkups. We excluded those who had a high risk of frailty and disability at baseline, which could mean that the participants who were sufficiently independent were more likely to have participated in the follow-up survey. This could have led to an underestimation of the effects of the assessed health problems and future risks. Besides, fewer participants had a relatively small degree of housing damage compared with those with other degrees of damage. Since a small degree of housing damage included a broad range of destruction, from moderate- to small-scale partially destroyed to not destroyed but flooded, it is possible that some survivors living these areas moved voluntarily and did not participate in the follow-up survey. Further studies with more detailed information on the participants could clarify the different characteristics and risks between participants and non-participants in the GEJE study.

The GEJE occurred in highly populated areas in Japan with declining populations and birthrates, as well as rapidly aging populations. Many of the survivors lost property and land, were disconnected from existing communities, and were cut off from the reconstruction and adaptation of new communities. Although our results are limited in terms of their ability to be generalized, preventive interventions in accordance with residential characteristics are expected to be one of the compelling practical examples for the prevention of frailty and disability in Japan’s aging society in the future.

The residential characteristics of the survivors continue to change year by year. Given the difficulty of changing the lifestyle habits of the elderly, it is thought that lifestyle factors are directly related to current living situations and the surrounding environment, as well as the original lifestyle before the disaster, rather than to housing damage itself. On the other hand, the damage experienced and the magnitude of damage itself continue to affect the psychological health of survivors over an extended period, especially in men. Those who could not recover from the deterioration in lifestyle and psychosocial factors after the disaster, even if they had a good lifestyle beforehand, may have had a higher risk for frailty.

## Conclusions

The results of the present study suggest that lifestyle and psychosocial factors associated with frailty risk differ by sex, the degree of housing damage, and residential status. The factors identified in this study are expected to be useful for the early detection and prevention of frailty among survivors in disaster-affected areas. More detailed information and additional preventive interventions based on residential characteristics may be important for the prevention of frailty among elderly disaster survivors in the future.

## Additional files

Additional file 1: **Table S1.** The Kihon Checklist in Japan. The checklist included a multidimensional 20-item index concerning lifestyle, motor abilities, nutrition, oral function, seclusion, and forgetfulness. (PDF 130 kb)

Additional file 2: **Table S2–1.** Factors associated with frailty (1 point increase) among male elderly survivors in the RIAS study, 2011–2015. (PDF 160 kb)

Additional file 3: **Table S2–2.** Factors associated with frailty (1 point increase) among female elderly survivors in the RIAS study, 2011–2015. These two tables were the results from a linear regression in the linear regression models. (PDF 155 kb)

## Abbreviations

95% CI: 95% Confidential Interval
BMI: Body mass index
GEJE: The Great East Japan Earthquake
HbA1c: Glycosylated hemoglobin
JDS: Japan diabetes society
K6: Kessler’s 6-item psychological distress scale
KCL: The Kihon Checklist
LTCI: Certification of long-term care insurance
METs: Metabolic equivalents
NGSP: The National Glycohemoglobin Standardization Program
OR: Odds Ratio
RIAS: The Research project for prospective Investigation of health problems Among Survivors of the GEJE
SRH: Self-rated health
TC: Total cholesterol level
