# Supplementary material for: Association between health risks and frailty in relation to the degree of housing damage among elderly survivors of the great East Japan earthquake
Source: BMC Geriatr. 2018 Jun 4;18:133. doi: 10.1186/s12877-018-0828-x (PMC6001143; doi:10.1186/s12877-018-0828-x)
Supplement: Supplementary file 1 — Table S1. The Kihon Checklist in Japan. The checklist included a multidimensional 20-item index concerning lifestyle, motor abilities, nutrition, oral function, seclusion, and forgetfulness. (PDF 130 kb) [file 12877_2018_828_MOESM1_ESM.pdf]

Table S1. the Kihon Checklist in Japan.

Domain	Question	Yes	No
Lifestyle	Do you normally travel by bus or train by yourself?	0	1
	Do you go out and by daily necessities by yourself?	0	1
	Do you manage your own deposits and savings at the bank?	0	1
	Do you often go out to visit your friends?	0	1
	Do you consult with your family or friends about their problems?	0	1
Motor abilities	Are you able to go upstairs without holding rail or wall?	0	1
	Are you able to stand up from the chair without any aids?	0	1
	Are you able to keep walking for about 15 minutes?	0	1
	Have you fallen during the past year?	1	0
	Do you worry about falling down?	1	0
Nutrition	Have you lost more than 2～3 kg in the past 6 months?	1	0
	Body mass index less than 18.5 kg/m^2	1	0
Oral function	Compared with six months ago, do you have difficulty in eating hard food?	1	0
	Do you choke when you drink tea or soup?	1	0
	Do you often feel your mouth dry?	1	0
Seclusion	Do you go out more than once in a week?	0	1
	Compared with last year, do you go out less often?	1	0
Forgetfulness	Do people around you say you repeat the same thing and have become forgetful?	1	0
	Do you make phone calls by yourself?	0	1
	Do you find yourself not knowing today's date?	1	0
