# Supplementary material for: Association between health risks and frailty in relation to the degree of housing damage among elderly survivors of the great East Japan earthquake
Source: BMC Geriatr. 2018 Jun 4;18:133. doi: 10.1186/s12877-018-0828-x (PMC6001143; doi:10.1186/s12877-018-0828-x)
Supplement: Supplementary file 2 — Table S2–1. Factors associated with frailty (1 point increase) among male elderly survivors in the RIAS study, 2011–2015. (PDF 160 kb) [file 12877_2018_828_MOESM2_ESM.pdf]

Table S2-1. Factors associated with frailty (1 point increase) among male elderly survivors in the RIAS study, 2011-2015.

Variable	***All participants in the present study.***		***By the degree of housing damage***						***By residential status*** [b]					
			**No**		**Partial**		**Extensive**		**No displaced**		**Temporary**		**Other residence**	
Number	1025		448		172		405		603		176		246	
	Adjusted coefficient (SE) [a]	***P-value***												
BMI, underweight	1.131 (0.548)	*0.039*	1.248 (0.887)	*0.160*	0.854 (1.027)	*0.406*	0.927 (0.629)	*0.141*	1.076 (0.721)	*0.136*	3.205 (0.221)	<.001	0.833 (0.732)	*0.255*
Current drinkers	-0.118 (0.067)	*0.081*	-0.212 (0.101)	*0.037*	-0.007 (0.161)	*0.965*	-0.094 (0.107)	*0.380*	-0.129 (0.087)	*0.138*	-0.168 (0.169)	0.320	-0.106 (0.133)	*0.425*
Physical inactivity	0.275 (0.058)	*<.001*	0.274 (0.089)	*0.002*	0.326 (0.134)	*0.016*	0.273 (0.092)	*0.003*	0.265 (0.074)	*<.001*	0.213 (0.146)	0.145	0.344 (0.112)	*0.002*
Sedentary lifestyle	0.259 (0.067)	*<.001*	0.270 (0.102)	*0.008*	0.174 (0.159)	*0.272*	0.253 (0.105)	*0.016*	0.297 (0.088)	*0.001*	0.225 (0.160)	0.158	0.164 (0.127)	*0.198*
Poor dietary diversity	0.114 (0.050)	*0.023*	0.098 (0.078)	*0.210*	0.108 (0.119)	*0.362*	0.121 (0.080)	*0.131*	0.086 (0.064)	*0.175*	-0.074 (0.142)	0.604	0.282 (0.098)	*0.004*
Poor self-rated health	0.857 (0.116)	*<.001*	0.877 (0.170)	*<.001*	0.773 (0.244)	*0.002*	0.851 (0.189)	*<.001*	0.862 (0.147)	*<.001*	1.043 (0.297)	0.001	0.709 (0.224)	*0.002*
Standard of living, difficult	0.192 (0.060)	*0.002*	0.253 (0.099)	*0.011*	0.239 (0.119)	*0.044*	0.121 (0.099)	*0.224*	0.244 (0.078)	*0.002*	0.005 (0.167)	0.975	0.224 (0.120)	*0.062*
Psychological distress	0.630 (0.207)	*0.002*	0.099 (0.319)	*0.756*	0.041 (0.273)	*0.881*	1.193 (0.333)	*<.001*	0.079 (0.213)	*0.710*	1.475 (0.439)	0.001	0.959 (0.484)	*0.048*
Poor social networks	0.360 (0.065)	*<.001*	0.307 (0.100)	*0.002*	0.267 (0.136)	*0.051*	0.442 (0.111)	*<.001*	0.298 (0.081)	*<.001*	0.306 (0.174)	0.080	0.532 (0.133)	*<.001*

BMI: Body Mass Index; SE: Standard Error

Nonsignificant variables are not displayed in table.

[a] Adjusted for age, body mass index (underweight: <18, overweight: ≥ 25 vs. normal: 18-25 kg/m^2), diabetes mellitus (yes vs no), current drinkers (yes vs no), physical activity (inactivity: <23METs·hour/week vs. ≥ 23METs·hour/week), sedentary lifestyle (yes vs. no), poor dietary diversity (yes vs. no), poor self-rated health (yes vs. no), standard of living (difficult vs. acceptable), psychological distress (yes vs. no), and poor social networks (yes vs. no).

[b] To avoid misclassification of participants' current situation, we continued updating each person's residential status throughout follow-up using data from repeated questionnaires.
