# Supplementary material for: Association between health risks and frailty in relation to the degree of housing damage among elderly survivors of the great East Japan earthquake
Source: BMC Geriatr. 2018 Jun 4;18:133. doi: 10.1186/s12877-018-0828-x (PMC6001143; doi:10.1186/s12877-018-0828-x)
Supplement: Supplementary file 3 — Table S2–2. Factors associated with frailty (1 point increase) among female elderly survivors in the RIAS study, 2011–2015. These two tables were the results from a linear regression in the linear regression models. (PDF 155 kb) [file 12877_2018_828_MOESM3_ESM.pdf]

Table S2-2. Factors associated with frailty (1 point increase) among female elderly survivors in the RIAS study, 2011-2015.

Variable	*All participants in the present study.*		*By the degree of housing damage*						*By residential status* [b]					
			**No**		**Partial**		**Extensive**		**No displaced**		**Temporary**		**Other residence**	
Number	1236		555		175		506		694		262		280	
	Adjusted coefficient (SE) [a] *P-value*													
BMI, underweight	0.521 (0.154)	*0.001*	0.817 (0.212)	*<.001*	0.434 (0.458)	*0.343*	0.255 (0.221)	*0.250*	0.739 (0.211)	*0.001*	0.746 (0.330)	*0.024*	-0.007 (0.247)	*0.976*
BMI, overweight	0.178 (0.068)	*0.009*	0.249 (0.100)	*0.013*	-0.048 (0.165)	*0.772*	0.181 (0.110)	*0.098*	0.202 (0.089)	*0.024*	0.240 (0.161)	*0.137*	0.046 (0.133)	*0.731*
Diabetes mellitus	0.146 (0.117)	*0.211*	0.095 (0.178)	*0.593*	0.144 (0.243)	*0.555*	0.186 (0.191)	*0.330*	0.132 (0.148)	*0.373*	0.181 (0.252)	*0.474*	0.097 (0.280)	*0.730*
Sedentary lifestyle	0.378 (0.062)	*<.001*	0.437 (0.100)	*<.001*	0.470 (0.182)	*0.010*	0.325 (0.088)	*<.001*	0.402 (0.092)	*<.001*	0.580 (0.134)	*<.001*	0.186 (0.103)	*0.072*
Poor dietary diversity	0.112 (0.050)	*0.026*	0.112 (0.072)	*0.122*	0.113 (0.128)	*0.381*	0.108 (0.084)	*0.201*	0.111 (0.064)	*0.083*	0.038 (0.115)	*0.745*	0.174 (0.115)	*0.132*
Poor self-rated health	0.704 (0.089)	*<.001*	0.668 (0.151)	*<.001*	0.511 (0.166)	*0.002*	0.835 (0.136)	*<.001*	0.576 (0.116)	*<.001*	0.956 (0.220)	*<.001*	0.761 (0.172)	*<.001*
Standard of living, difficult	0.231 (0.055)	*<.001*	0.303 (0.092)	*0.001*	0.408 (0.144)	*0.005*	0.137 (0.079)	*0.084*	0.307 (0.082)	*<.001*	0.223 (0.114)	*0.050*	0.127 (0.098)	*0.193*
Psychological distress	0.537 (0.112)	*<.001*	0.756 (0.209)	*<.001*	0.489 (0.292)	*0.095*	0.424 (0.143)	*0.003*	0.685 (0.192)	*<.001*	0.261 (0.180)	*0.147*	0.641 (0.188)	*0.001*
Poor social networks	0.212 (0.053)	*<.001*	0.174 (0.078)	*0.026*	0.406 (0.139)	*0.004*	0.203 (0.084)	*0.016*	0.286 (0.069)	*<.001*	0.141 (0.114)	*0.216*	0.136 (0.117)	*0.246*

BMI: Body Mass Index; SE: Standard Error

Nonsignificant variables are not displayed in table.

[a] Adjusted for age, BMI (underweight: <18, overweight: ≥ 25 vs. normal: 18-25 kg/m^2), diabetes mellitus (yes vs no), sedentary lifestyle (yes vs. no), poor dietary diversity (yes vs. no), poor self-rated health (yes vs. no), standard of living (difficult vs. acceptable), psychological distress (yes vs. no), and poor social networks (yes vs. no).

[b] To avoid misclassification of participants' current situation, we continued updating each person's residential status throughout follow-up using data from repeated questionnaires.
